# A Hybrid Algorithm for Missing Data Imputation and Its Application to Electrical Data Loggers

**DOI:** 10.3390/s16091467

**Published:** 2016-09-10

**Authors:** Concepción Crespo Turrado, Fernando Sánchez Lasheras, José Luis Calvo-Rollé, Andrés-José Piñón-Pazos, Manuel G. Melero, Francisco Javier de Cos Juez

**Affiliations:** 1 Maintenance Department, University of Oviedo, San Francisco 3, Oviedo 33007, Spain; ccrespo@uniovi.es; 2 Department of Construction and Manufacturing Engineering, University of Oviedo, Campus de Viesques, Gijón 33204, Spain; 3 Departamento de Ingeniería Industrial, University of A Coruña, A Coruña 15405, Spain; jlcalvo@udc.es (J.L.C.-R.), andres.pinon@udc.es (A.-J.P.-P.); 4 Electrical Engineering Department, University of Oviedo, Campus de Viesques, Gijón 33204, Spain; melero@uniovi.es; 5 Prospecting and Exploitation of Mines Department, University of Oviedo, Oviedo 33004, Spain; fjcos@uniovi.es

**Keywords:** missing data imputation, multivariate imputation by chained equations (MICE), Mahalanobis distances, Self-Organized Maps Neural Networks (SOM), Adaptive Assignation Algorithm (AAA), Multivariate Adaptive Regression Splines (MARS), quality of electric supply, voltage, current, power factor

## Abstract

The storage of data is a key process in the study of electrical power networks related to the search for harmonics and the finding of a lack of balance among phases. The presence of missing data of any of the main electrical variables (phase-to-neutral voltage, phase-to-phase voltage, current in each phase and power factor) affects any time series study in a negative way that has to be addressed. When this occurs, missing data imputation algorithms are required. These algorithms are able to substitute the data that are missing for estimated values. This research presents a new algorithm for the missing data imputation method based on Self-Organized Maps Neural Networks and Mahalanobis distances and compares it not only with a well-known technique called Multivariate Imputation by Chained Equations (MICE) but also with an algorithm previously proposed by the authors called Adaptive Assignation Algorithm (AAA). The results obtained demonstrate how the proposed method outperforms both algorithms.

## 1. Introduction

Currently, the importance of problems due to harmonics in electric networks is growing. This fact is due to the increase in the amount of non-linear loads. The two main problems related to harmonics are the overheating of conductors due to the skin effect and the activation of automatic breakers, which produce problems for supply continuity. Additionally, distortion of the voltage waveform may cause the malfunction of some devices. The monitoring of harmonics in real time is required to control them.

Another common problem in electrical networks is the imbalance between phases. This is usually caused by a bad load distribution between phases and provokes a high current return displayed by the neutral, as it has to compensate for the gap existing at the centre of the scheme vectors. 

Electricity quality is an important issue that is present in the following variables: voltage, current, frequency anomalies, etc. The quality affects all devices connected to the power network, causing failure of the systems or disability [1]. Currently, an electric system is analyzed in terms of efficiency, stability and optimization to obtain better quality of the system [2]. With the aim of reducing the issues and improving electricity quality, science and technology are evolving to mitigate problems and overcome the problems mentioned above [3].

Different studies in this field have been performed: for instance, a novel power quality deviation parameter based on principal curves is presented in [4]. In [5], a review of the signal processing and intelligent techniques and methods employed in the self-classification of the events of power quality and the influence of noise on the recognition and classification of perturbations has been made. [6] describes a device capable of labelling, recognizing, and quantifying energy and power quality perturbation. An intelligent device for high-resolution frequency measuring that agrees with the common indicator standards is shown in [7]; it is used for electricity quality monitoring and control. Furthermore, [8] exposes a communication infrastructure created to obtain consistent data delivery at low cost, with the aim to prevent the difficulties of the power quality monitoring service.

Monitoring the main electrical variables in electric systems in some buildings might be interesting. Therefore, monitoring is useful for the control with the objective of balancing the loads of a building, thus reducing the consumption of the electric energy of the building by decreasing the remaining consumption (during non-working hours). The analysis of the electric system in buildings is useful for determining the optimized rates. Furthermore, it is also useful for the analysis of supply issues that can affect the different loads, which are caused by a lack of balance or harmonics, analysis of the energy quality and preventing incidents as a result of poor signal quality. Finally, the analysis of the building operation and its efficiency study might be of interest when accounting for the dependency of the people who use it, area in use, installed power, etc.

During the data-collection process, it is likely that a small amount of the information retrieved may be lost. In these cases, missing data imputation algorithms must be applied because the substitution of missing data with zeros is not acceptable. The present research evaluates a new imputation method that is able to predict the value of any missing data in the sensor devices that are used in this research for the recording of electrical variables. The new algorithm is based on Self-Organized Maps Neural Networks and Mahalanobis distances and hybridizes them with the algorithm called the Adaptive Assignation Algorithm (AAA). The results obtained are benchmarked with those given by the AAA and multivariate imputation by chained equations (MICE) [9].

The rest of the paper is arranged as follows: Section 2 describes the measurement equipment and the database, Section 3 details the proposed algorithm and how its performance is measured and compared. Section 4 presents the results obtained and its comparison with other algorithms. Finally, the conclusions are drawn in Section 5.

## 2. Materials and Methods

### 2.1. Measurement Equipment

**Figure 1:**
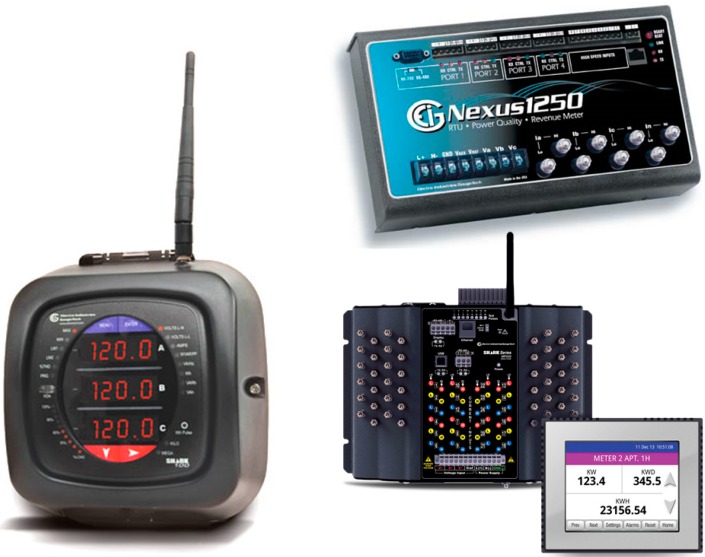
Equipment (SK-100/200 on the left, Nexus1250 on the right top and MP200 on the right bottom; source: Electro Industries/GaugeTech, Westbury, New York—USA) [10].

In this section, the specific power quality measurement devices that are employed in this work are described (Figure 1). The next measurements are common to all them, namely: Energy Output or Input (ENERGY), Reactive Energy Output or Input (R-ENERGY), Apparent Energy (A-ENERGY), Power Factor (P-F), Power Output or Input (POW), Reactive Power (R-POW), Apparent Power (A-POW), Voltage from Line to Line (VLL), Voltage from Line to Neutral (VLN), Current by line (I), and Frequency (FQ). The accuracy of each electrical measurement for all devices used is shown in Table 1. Note that all indicated percentage values refer to the obtained percentage.

All mentioned measurements can be performed by the four devices used in the present work.

The four devices have additional features. Shark 100, Shark 200 and Shark MP200 incorporate V-Switch technology, which allows the operator to add new functions to the devices using programming commands at any time after its installation. In the case of the Nexus 1252 device, it is possible to add isolated input/output modules and software options for additional functions. All of them have communication capabilities (some optional) as Modbus or DNP 3.0 (Distributed Network Protocol) protocols by an RS485 port, 10/100BaseT Ethernet capabilities or IrDA port. A deep analysis of features of each device is made in [10].

### 2.2. The Data Description

In this paper, the next dataset, which includes measurements of variables from an electrical power supply of an edifice, has been used.

- Three variables of each phase current
- Three variables of voltage from phase to phase
- Three variables of voltage from phase to neutral
- Average power factor

Between the 27 November 2014 at 18:45 and the 31 May 2015 at 23:45, the data set was logged, with an interval of 15 min.

A building called Severo Ochoa, in honour of the Novel Prize winner, was used in this work for the dataset. The University of Oviedo (Spain) is the owner of this building, which has a total area of 8.150 m^2^, distributed over two basement levels and five floors; a total of 78 employees work in it. The ITS (Information Technology Services) of the University of Oviedo are also located in this edifice. The equipment of the ITS is distributed across server rooms and scientific laboratories. This equipment has to be supplied by a good quality power network at all times. The laboratory equipment mentioned above includes electron microscopes, NMR spectrometers, X-ray diffractometers, etc. The energy consumption is 190.572 KWh per day, on average. The data set detailed here was already employed by the authors in previous research [9].

The equipment mentioned above, and the building services, incorporate devices such as UPS (Uninterruptible Power Supply), VSD (Variable Speed Drive) and inductive and capacitive loads in switching mode. These electronic circuits are nonlinear loads, and all of them can create harmonic distortion in the power line. The harmonic distortion in the distribution system is caused by the harmonic currents flowing in the electronic loads.

## 3. Methodology

The data set employed in this research has a total of 17,763 samples that correspond to the period of time referred to in the description of the data. A process of random data deletion was performed using this data set.

The new algorithm presented in this paper hybridizes the Self-Organized Maps Neural Networks methodology with the Mahalanobis distances. The hybrid method obtained is combined with an algorithm already presented in this journal by the authors, called AAA [9], based on Multivariate Adaptive Regression Splines. The proposed methodology is new and its performance is even better than the one referenced and presented in a previous paper when applied to the same database. This method is considered a hybrid method because it combines well-known pattern recognition and machine learning methodologies in a hybrid model that is able to impute missing data [11,12].

The performance of the proposed new methodology, in comparison with AAA and MICE, has been evaluated using the mean absolute error (MAE) and the root mean square error (RMSE). They are very common metrics in forecasting research [13,14]. The reason why, in the present research, both are employed is their complementarity. The purpose of the MAE is the measurement of the average magnitude of the error in a set of forecasts without considering their direction while the RMSE is employed for its ability to describe uniformly distributed errors [13]. A more detailed explanation including the formulas employed can be found in [9]

Let us assume that we have a dataset formed by c different variables $v_{1},\;v_{2},\;\ldots ,\;v_{c}$ that are the columns of a data matrix whose total number of rows is r. The algorithm is applied via the following steps.

### 3.1. Creation of a New Matrix with Missing Values from the Original Data Set

This step of the algorithm is not required when it is applied to a data set in which missing data are going to be imputed, but it is mandatory in the present research to validate the algorithm by using a complete data set.

Let A be the original matrix (rxc) of r rows and c columns. As a first step and to obtain a matrix with a certain amount of missing data, a proportion of p elements in the matrix is removed. Let B be the new (rxc) matrix, with a proportion p of missing elements. The removal is performed completely at random; therefore, the type of imputation that is going to be tested to determine the performance of the algorithm is the one known as missing completely at random (MCAR).

### 3.2. Creation of the Reduced Matrix

A new matrix in which all the rows with missing data are removed is created. This new matrix is called *B^red^*. Although the number of rows *s* (*s* ≤ *r*) of this matrix will change depending on the matrix that is going to be imputed, in those cases like the one presented in this algorithm in which the removal of data has been performed completely at random and in a proportion p, the number of remaining rows u will be represented by the following formula:

(1) $$u=r\times {\left(1-p\right)}^{c},$$

where:

p: proportion of missing data considered;
r: number of rows of the original matrix;
c: number of columns of the data matrix;
Afterwards the $B^{red}$ matrix is normalized.

### 3.3. Determination of the Director Vectors by Means of Self-Organized Maps Neural Networks

The Self-Organized Maps (SOM) Neural Network is a type of unsupervised neural-network algorithm whose main application is related to the visualization and interpretation of large dimensional data sets [15].

These types of maps are used to represent all the available observations (data vectors), with an optimized accuracy, by means of a reduced set of models. This is the reason why this technique has been chosen in the present research.

Let N be the dimension of the n director vectors X(t) ∈ $R^{n}$, t = 1, 2, …, n, where each sample vector is identified by a label. The two-dimensional output layer of the SOM map contains a rectangular mesh of k = 1, …, x_dim_ × y_dim_ nodes. Each one of these nodes is employed as a codebook vector W_k_ of dimension N. The calculus of the weight vectors is performed by using the following algorithm [16].

For a certain amount of iterations, follow the steps detailed below:

1. Choose one sample vector X(t) at random;
2. Search for the nearest weight vector $W_{c}:||X-W_{c}||=min_{j}||X-W_{j}||;$
3. Update the weights $W_{i}$ by means of the following rule:
  (2) $$W_{i}\left(t+1\right)=W_{i}\left(t\right)+h_{ci}\left(t\right)\cdot \left[X\left(t\right)-W_{i}\left(t\right)\right],$$

where $h_{ci}\left(t\right)$ is the neighbour function, which, in the case of the present research and is being very common in the literature [15], is of the Gaussian type:

(3) $$h_{ci}\left(t\right)=\alpha \left(t\right)\cdot \exp\left(\frac{-||W_{c}-W_{i}||}{2\cdot \sigma^{2}\left(t\right)}\right).$$

Weight of neurons lying in the neighbourhood $h_{ci}\left(t\right)$ of the winning neuron is moved closer to X(t). The learning rate α(t)∈[0,1] decreases monotously as the number of iterations increases, σ(t) determining that the radius of the neighbourhood also decreases monotonically. After many iterations and the slow reduction of α(t) and σ(t), the neighbourhood covers only a single node and the map is formed. Please note that those neurons, whose weights are closer in the parameter space W, are also closer on the mesh. After this process, the director vectors obtained are denormalized. The number of director vectors chosen to create the Self-Organized Map in the case of the present algorithm is related to the number of rows in the $B^{red}$ matrix. Let u be the number of rows in the matrix $B^{red}$; the total amount of director vectors will be a range of values d=e·u / e∈[0.05, 0.8]; the reason for this range of values, empirically found, will be explained in the results sections.

### 3.4. Finding the Closest Director Vectors by Means of Mahalanobis Distances

The Mahalanobis distance is a well-known, non-Euclidean distance measure based on correlations between variables [17]. These correlations allow for the identification and analysis of different patterns. This measure is a useful way of determining the similarity of an unknown sample set to a known one, and, in the present research, it is used to compare each one of the rows of the data matrix with missing data with all the director vectors. It can be defined by the following formula:

(4) $$d_{A}\left(x_{1},x_{2}\right)=\sqrt{{\left(x_{1}-x_{2}\right)}^{T}\cdot A\cdot \left(x_{1}-x_{2}\right),}$$

where $x_{1}$ and $x_{2}$ represent the sets of variables of two different rows of the data matrix, and $A\;\in R^{nxn}$ is a positively semi-definite matrix that represents the inverse of the covariance matrix of class {I}. By means of the eigenvalue decomposition, A can be decomposed into $A=W\cdot W^{T}$.

In the case of the present algorithm, the Mahalanobis distance of each vector row with two or more missing data points to all the director vectors is calculated. Please note that, in order to make this operation possible, all those variables with missing data in the row that come from the data matrix are removed in the director vector. The director vector with the lowest Mahalanobis distance value is selected and those missing variables in this row of the data matrix are filled using the values present in the corresponding row of the director vector.

Finally, the original matrix is reconstructed and the value of the missing data of those rows with only one or two missing data points are imputed by means of the AAA algorithm. As it has already been stated, this algorithm was presented in a previous work [9] published in this journal. The referenced algorithm is based on a multivariate non-parametric technique called Multivariate Adaptive Regression Splines (MARS) [18,19,20,21].

## 4. Results and Discussion

In this section, the results of the Hybrid Adaptive Assignation Algorithm (HAAA) are presented and compared with those of the AAA and MICE. The test was performed using the MCAR methodology, deleting 10%, 15% and 20% of the information. This process was repeated five times. The performances of the three algorithms were compared based on the MAE and RMSE metrics. The results of all the interactions performed are presented. To simplify the comparisons, the results that use the same original MCAR subsets are presented in the same table. The way in which results are presented is the same as the one that was employed in previous research, in which the performance of the AAA algorithm was analysed [9]. Each table also contains the average values of the five replications. Table 2 contains the RMSE values of the MICE, AAA and HAAA algorithms when applied to a database with 10% of the data missing. As can be observed in this table, for the variables of voltage, intensity and power factor employed in this research, the RMSE values obtained by the new algorithm are considerably lower than those obtained by using the AAA and MICE methods. In the case of 10% missing data, Table 2, the variable in which the RMSE is reduced to a lesser amount receives a 15% reduction, while the average reduction of all variables is 62%. For the case of 15% missing data, Table 3, the results are very similar, obtaining at least a reduction of the RMSE of 12% and an average reduction of 46%. Additionally, for the case of 20% missing data, Table 4, the results are equivalent, with a minimum 18% reduction of the RMSE value and an average of 48%.

The results obtained when the MAE metric is applied to the three algorithms are equivalent. Table 5 shows the results obtained using the MAE metric for 10% missing data, while Table 6 does the same for 15% and Table 7 for 20%. When the algorithm proposed is compared with AAA in the case of 10% missing data, the average of improvement regarding the MAE metric is 35%, with a minimum value of 10%. For the case of 15% missing data, the average improvement of the MAE is 29%, with a minimum of an 8% improvement in one of the variables. When the amount of missing data is 20%, the average improvement of the referenced metric is 42%, with a minimum amount of 13%.

Although the overall performance of the new algorithm has already been evaluated using MCAR data, from the point of view of the authors, there are a couple of situations in which the information is not missing completely at random and are of great interest for electrical measurements. These are as follows:

- The case in which there is correlation in the missingness of data: one possible situation when working with electrical data would be when all the missing information corresponds to the same phase. In order to simulate this kind of failure, five new data sets with a 20% of missing data were created. Each phase is represented by means of four different variables: one variable of phase current, two variables of voltage from phase to phase and one variable of voltage from phase to neutral. It means that each row with missing incomplete information has four missing variables or, in other words, that only 5% of the total of rows will have missing data. In the referred rows, randomly selected, the information of the variables of one of the phases was removed. It means that, for example, when information for variable $V_{an}$ is missing, it is also missing the information of variables, $V_{ab}$, $V_{ca}$ and $I_{a}$. The results obtained are presented in Table 8 and Table 9. As it can be observed, the performance of the HAAA algorithm is worse than in the MCAR case, but it outperforms both MICE and AAA.
- The case in which most of the missing data correspond to a certain subset of variables. In order to simulate this kind of failure, five new datasets with a 90% of missing data in a single variable were created. In each dataset, a proportion of 90% elements in one single column were removed, leaving the rest of the variables with their original values. As it can be seen in Table 10, the imputation accuracy for all the algorithms decreased significantly. This was expected in such an unfavourable situation; however, it is possible to ascertain, as both algorithms HAAA and AAA considerably outperform the algorithm of reference MICE, HAAA being the one with the best results.

## 5. Conclusions

The improvement of power quality has become a necessity as the presence of power electronics in today’s grids has been increasing in the last decades. Due to this problem, network monitoring with the help of real-time data collection devices is helpful. In this context, the availability of missing data imputation techniques is required.

This research presents a new algorithm and compares it with another algorithm proposed in a previous paper by the authors and also with a well-known missing data imputation algorithm. Although the algorithm presented in this paper outperforms the others, as the previous methods to which it is compared, it also has some limitations that must be taken into account. As those proposed before, our algorithm would have imputation problems in those cases in which most of the missing data belonged to the same variable or were concentrated in a certain subset of variables instead of distributed among all the variables of the data set. Currently, the authors continue to develop hybrid algorithms that would improve the results of existing algorithms when they have to address this type of issue. Finally, the missing data imputation in the time-frequency domain will also be explored in future works.

## Figures and Tables

**Table 1:** Device precision. POW: power, R-POW: reactive power, A-POW: active power, A-ENERGY: active energy, VLN: voltage line to neuter, VLL: voltage line to line, I: current, PF: power factor, FQ: frequency.

Variable	Units	MP200	NEXUS 1252		SK-200	SK-100
		(%)	200 mili Seg (%)	1 s (%)	(%)	(%)
POW	W	0.5	0.1	0.06	0.2	0.2
ENERGY	W·h	0.5	N/A	0.04	0.2	0.2
R-POW	VARs	1.0	0.1	0.08	0.2	0.2
R-ENERGY	VAR·h	1.0	N/A	0.08	0.2	0.2
A-POW	VA	1.0	0.1	0.1	0.2	0.2
A-ENERGY	VA·h	1.0	N/A	0.08	0.2	0.2
VLN	V/KV	0.3	0.1	0.05	0.1	0.1
VLL	V/KV	0.5	0.1	0.05	0.2	0.1
I	A/KA	0.3	0.1	0.025	0.1	0.1
PF	0.5 to 1	1.0	0.1	0.08	0.2	0.2
FQ (*)	Hz	±10^−2^ *	3.10^−2^ *	1.10^−2^ *	±3.10^−2^ *	1.10^−2^ *

* Accuracy in Hz.

**Table 2:** RMSE obtained with 10% missing data using MICE, AAA and the newly proposed algorithm, HAAA. RMSE: root mean square error, MICE: multivariate imputation by chained equations, AAA: adaptive assignation algorithm, HAAA: hybrid adaptive assignation algorithm, Van: voltage line a to neuter, Vbn: voltage line b to neutre, Vcn: voltage line c to neutre, Vab: voltage line a to b, Vbc: voltage line b to c, Vca: voltage line c to a, Ia: current line a, Ib: curren line b, Ic: current line c.

	**RMSE MICE 10% Missing Data**									
**ID#**	**Van**	**Vbn**	**Vcn**	**Vab**	**Vbc**	**Vca**	**Ia**	**Ib**	**Ic**	**PF**
1	20.8398	34.3633	21.9653	30.9830	36.0714	48.0547	0.2653	0.7807	1.6655	0.0030
2	18.7495	31.6307	24.1316	31.0080	37.0614	48.1173	0.7918	0.3262	1.5742	0.0017
3	18.8833	30.4312	24.0313	34.9034	30.8320	47.8822	0.1946	0.1122	1.6727	0.0027
4	17.3260	32.0759	21.2884	31.8996	31.5952	48.7585	0.9634	0.6172	1.8090	0.0030
5	20.0333	34.4660	22.1438	32.1480	32.9437	47.7413	0.7631	0.1324	1.4919	0.0028
**average**	19.1664	32.5934	22.7121	32.1884	33.7007	48.1108	0.5956	0.3937	1.6427	0.0026
	**RMSE AAA Algorithm 10% Missing Data**									
**ID#**	**Van**	**Vbn**	**Vcn**	**Vab**	**Vbc**	**Vca**	**Ia**	**Ib**	**Ic**	**PF**
1	1.0583	1.7376	1.1078	1.6251	1.0612	1.8042	0.1318	0.1478	0.1514	0.0030
2	1.0228	1.6687	1.2186	1.4223	2.0596	1.7124	0.1307	0.1749	0.1304	0.0020
3	0.9641	1.5329	1.2044	2.0471	1.9560	1.7213	0.1365	0.1985	0.1172	0.0015
4	0.9328	1.6923	1.1531	1.8030	1.8338	1.8457	0.1845	0.1581	0.1340	0.0021
5	1.0473	1.7783	1.1100	1.7025	1.5521	1.8885	0.1368	0.1374	0.2158	0.0024
**average**	1.0050	1.6819	1.1588	1.7200	1.6925	1.7944	0.1441	0.1633	0.1498	0.0022
	**RMSE New Algorithm 10% Missing Data**									
**ID#**	**Van**	**Vbn**	**Vcn**	**Vab**	**Vbc**	**Vca**	**Ia**	**Ib**	**Ic**	**PF**
1	0.6029	0.6657	0.6165	0.0384	0.0218	0.0265	0.0866	0.0632	0.0863	0.0009
2	0.5663	0.6128	0.5283	0.0270	0.0558	0.0503	0.0599	0.0896	0.0687	0.0008
3	0.5789	0.6526	0.5457	0.0690	0.0497	0.0479	0.0768	0.0652	0.0687	0.0061
4	0.5264	0.5965	0.6352	0.0344	0.0383	0.0374	0.0608	0.0624	0.0609	0.0007
5	0.5853	0.5110	0.5568	0.0603	0.0354	0.0316	0.0612	0.0745	0.0523	0.0009
**average**	0.5720	0.6077	0.5765	0.0458	0.0402	0.0387	0.0691	0.0710	0.0674	0.0019

**Table 3:** RMSE obtained with 15% missing data using MICE, AAA and the newly proposed algorithm, HAAA.

	**RMSE MICE 15% Missing Data**									
**ID#**	**Van**	**Vbn**	**Vcn**	**Vab**	**Vbc**	**Vca**	**Ia**	**Ib**	**Ic**	**PF**
1	21.2798	34.9133	22.2953	36.3636	31.1154	44.9074	0.3129	1.2746	1.0503	0.0031
2	19.1895	31.9607	24.4616	37.2800	31.0974	45.0104	0.5060	1.3345	1.7062	0.0034
3	19.1033	30.7612	24.4713	37.1822	31.1052	44.6659	0.4039	1.2087	1.1608	0.0036
4	17.6560	32.4059	21.6184	37.1192	30.6655	45.9235	0.6803	1.9764	1.8724	0.0029
5	20.4733	35.1260	22.8038	37.4040	30.9877	44.5148	0.3754	1.1864	1.2194	0.0043
**average**	19.5404	33.0334	23.1301	37.0698	30.9942	45.0044	0.4557	1.3961	1.4018	0.0035
	**RMSE AAA Algorithm 15% Missing Data**									
**ID#**	**Van**	**Vbn**	**Vcn**	**Vab**	**Vbc**	**Vca**	**Ia**	**Ib**	**Ic**	**PF**
1	1.1133	1.8036	1.1298	0.0591	0.0650	0.1153	2.0629	2.1338	2.5664	0.0009
2	1.0778	1.6907	1.2406	0.0620	0.0629	0.1176	2.2728	1.5485	2.6213	0.0009
3	1.0081	1.5549	1.2374	0.0493	0.0656	0.1014	1.8102	1.9783	2.6717	0.0012
4	0.9658	1.7583	1.2191	0.0523	0.0393	0.1501	2.2242	1.5741	2.5330	0.0012
5	1.1023	1.8333	1.1320	0.0678	0.0533	0.0929	1.7768	2.1714	2.4560	0.0010
**average**	1.0534	1.7281	1.1918	0.0581	0.0572	0.1155	2.0294	1.8812	2.5697	0.0011
	**RMSE New Algorithm 15% Missing Data**									
**ID#**	**Van**	**Vbn**	**Vcn**	**Vab**	**Vbc**	**Vca**	**Ia**	**Ib**	**Ic**	**PF**
1	0.6689	0.6987	0.6715	0.0439	0.0262	0.0309	0.6936	0.6543	0.7210	0.0010
2	0.5883	0.6678	0.5943	0.0325	0.0580	0.0536	0.6579	0.7209	0.7350	0.0009
3	0.6449	0.6966	0.5787	0.0745	0.0541	0.0545	0.8358	0.7183	0.7421	0.0011
4	0.5814	0.6185	0.6792	0.0388	0.0427	0.0407	0.6739	0.6574	0.6531	0.0008
5	0.6403	0.5550	0.6008	0.0658	0.0409	0.0349	0.6336	0.7715	0.7050	0.0009
**average**	0.6248	0.6473	0.6249	0.0511	0.0444	0.0429	0.6990	0.7045	0.7112	0.0009

**Table 4:** RMSE obtained with 20% missing data using MICE, AAA and the newly proposed algorithm, HAAA.

	**RMSE MICE 20% Missing Data**									
**ID#**	**Van**	**Vbn**	**Vcn**	**Vab**	**Vbc**	**Vca**	**Ia**	**Ib**	**Ic**	**PF**
1	23.3518	36.9853	24.0713	43.6987	33.1613	50.9935	0.5688	0.6465	1.1420	0.0941
2	21.8535	34.3287	25.3496	30.8402	36.5679	53.5682	0.6665	0.5931	1.2083	0.0906
3	19.9913	33.1292	25.9513	40.3302	27.7061	53.0747	0.5814	0.5970	1.0409	0.0550
4	20.0240	33.5899	23.3944	30.7252	35.7307	51.8370	0.5847	0.3919	1.4901	0.0843
5	22.8413	36.0140	25.4678	45.1604	28.2537	48.6319	0.6590	0.5827	1.0313	0.1027
**average**	21.6124	34.8094	24.8469	38.1509	32.2839	51.6210	0.6121	0.5622	1.1825	0.0853
	**RMSE AAA Algorithm 20% Missing Data**									
**ID#**	**Van**	**Vbn**	**Vcn**	**Vab**	**Vbc**	**Vca**	**Ia**	**Ib**	**Ic**	**PF**
1	1.2317	1.4731	1.1482	2.2405	2.2522	2.8032	0.142	0.1537	0.1597	0.0058
2	1.2850	1.3387	1.2478	2.53918	1.6669	2.7101	0.1353	0.1772	0.1731	0.0038
3	1.1857	1.2352	1.2742	2.07656	2.1855	2.8789	0.1162	0.1459	0.155	0.0068
4	1.0546	1.2359	1.1375	2.4018	1.7221	2.6810	0.162	0.146	0.1259	0.0066
5	1.3687	1.4813	1.1392	1.98403	2.2898	2.6632	0.1196	0.1533	0.1304	0.0077
**average**	1.2251	1.3528	1.1894	2.2484	2.0233	2.7473	0.1350	0.1552	0.1488	0.0061
	**RMSE New Algorithm 20% Missing Data**									
**ID#**	**Van**	**Vbn**	**Vcn**	**Vab**	**Vbc**	**Vca**	**Ia**	**Ib**	**Ic**	**PF**
1	0.8761	0.9651	0.8787	0.4692	0.4224	0.4128	0.0978	0.0929	0.1123	0.0014
2	0.7659	0.7566	0.7127	0.3311	0.5584	0.6419	0.1118	0.1012	0.1252	0.0017
3	0.9113	0.9038	0.7267	0.7379	0.5989	0.5070	0.1341	0.1157	0.1076	0.0018
4	0.7294	0.8849	0.9456	0.4763	0.4598	0.4680	0.0953	0.1044	0.1112	0.0016
5	0.7587	0.8214	0.7192	0.6213	0.5170	0.3739	0.1125	0.1186	0.1066	0.0017
**average**	0.8083	0.8664	0.7966	0.5272	0.5113	0.4807	0.1103	0.1066	0.1126	0.0016

**Table 5:** MAE (mean absolute error) obtained with 10% missing data using MICE, AAA and the newly proposed algorithm, HAAA.

	**MAE MICE 10% Missing Data**									
**ID#**	**Van**	**Vbn**	**Vcn**	**Vab**	**Vbc**	**Vca**	**Ia**	**Ib**	**Ic**	**PF**
1	16.5255	27.4609	17.6382	31.0773	31.8032	39.9026	0.1725	0.2588	1.3358	0.0024
2	14.6229	24.1967	20.2526	33.6486	24.1749	40.8152	0.1744	0.2355	1.3055	0.0025
3	15.1412	23.9954	19.4134	26.2969	30.7039	41.4323	0.2113	0.2623	1.3377	0.0024
4	14.0678	25.6064	17.3741	34.8765	23.4809	37.3655	0.2512	0.2306	1.3785	0.0026
5	16.0730	27.5605	17.8741	27.4130	33.2976	35.7481	0.2334	0.1752	1.4786	0.0060
**average**	15.2861	25.7640	18.5105	30.6624	28.6921	39.0528	0.2086	0.2325	1.3672	0.0032
	**MAE AAA Algorithm 10% Missing Data**									
**ID#**	**Van**	**Vbn**	**Vcn**	**Vab**	**Vbc**	**Vca**	**Ia**	**Ib**	**Ic**	**PF**
1	0.8120	0.8681	0.7423	0.4379	1.3656	2.5745	0.1117	0.1210	0.9243	0.0059
2	0.8358	0.8545	0.7946	1.5311	1.2375	0.8110	0.1210	0.1228	1.0954	0.0062
3	0.8274	0.8546	0.7923	1.1356	1.4964	1.3782	0.1215	0.1272	0.9566	0.0095
4	0.8653	0.8651	0.7562	1.3314	1.4677	1.3657	0.1186	0.1222	1.0425	0.0085
5	0.9052	0.8561	0.7563	1.2364	1.2115	0.8277	0.1145	0.1177	0.9595	0.0120
**average**	0.8491	0.8597	0.7684	1.1345	1.3557	1.3914	0.1175	0.1222	0.9957	0.0084
	**MAE New Algorithm 10% Missing Data**									
**ID#**	**Van**	**Vbn**	**Vcn**	**Vab**	**Vbc**	**Vca**	**Ia**	**Ib**	**Ic**	**PF**
1	0.8192	0.7406	0.6883	0.3447	0.5175	1.0331	0.1047	0.1091	0.1338	0.0042
2	0.6789	0.6371	0.7580	0.5359	0.5140	1.0838	0.1163	0.0872	0.1410	0.0033
3	0.7080	0.6473	0.7381	0.4839	0.3278	1.0783	0.0923	0.1055	0.1387	0.0054
4	0.6946	0.7001	0.6387	0.5285	0.2821	0.8947	0.1224	0.0845	0.1253	0.0049
5	0.7616	0.7693	0.6695	0.1723	0.6028	1.0647	0.0926	0.1158	0.1217	0.0069
**average**	0.7325	0.6989	0.6985	0.4131	0.4488	1.0309	0.1057	0.1004	0.1321	0.0049

**Table 6:** MAE obtained with 15% missing data using MICE, AAA and the newly proposed algorithm, HAAA.

	**MAE MICE 15% Missing Data**									
**ID#**	**Van**	**Vbn**	**Vcn**	**Vab**	**Vbc**	**Vca**	**Ia**	**Ib**	**Ic**	**PF**
1	17.1855	27.7909	18.2982	32.3532	40.3426	31.4073	0.7919	0.9918	2.0018	0.0149
2	15.2829	24.4167	20.6926	24.3949	41.2552	34.0886	1.0895	0.9905	2.0695	0.0141
3	15.5812	24.6554	19.6334	31.0339	41.9823	26.7369	0.3773	0.6813	2.0927	0.0171
4	14.6178	26.0464	17.8141	23.7009	37.6955	35.2065	0.5732	0.5966	1.8180	0.0189
5	16.2930	27.8905	18.5341	33.9576	35.9681	27.6330	0.4885	1.2168	2.1226	0.0205
**average**	15.7921	26.1600	18.9945	29.0881	39.4488	31.0144	0.6641	0.8954	2.0209	0.0171
	**MAE AAA Algorithm 15% Missing Data**									
**ID#**	**Van**	**Vbn**	**Vcn**	**Vab**	**Vbc**	**Vca**	**Ia**	**Ib**	**Ic**	**PF**
1	0.9321	0.9516	0.8651	1.3788	1.4240	1.3866	0.0932	0.0952	0.0865	0.0063
2	0.9576	0.8613	0.9000	1.3569	1.3313	1.3687	0.0958	0.0861	0.09	0.0075
3	0.5774	0.8965	0.8652	1.3458	1.5144	1.3788	0.0577	0.0896	0.0865	0.0101
4	0.9626	0.8945	0.9513	1.3565	1.5227	1.3744	0.0963	0.0895	0.0951	0.0096
5	0.9342	0.9852	0.9806	1.3026	1.5278	1.3506	0.0934	0.0985	0.0981	0.0124
**average**	0.8728	0.9178	0.9125	1.3481	1.4640	1.3718	0.0873	0.0918	0.0912	0.0092
	**MAE New Algorithm 15% Missing Data**									
**ID#**	**Van**	**Vbn**	**Vcn**	**Vab**	**Vbc**	**Vca**	**Ia**	**Ib**	**Ic**	**PF**
1	0.7185	0.5040	0.6644	0.4819	0.5835	1.0881	0.0797	0.0851	0.0842	0.0072
2	0.5999	0.4390	0.7368	0.5861	0.5800	1.1278	0.0882	0.0699	0.0881	0.0056
3	0.6245	0.4420	0.7256	0.2459	0.3938	1.1113	0.0696	0.0839	0.0881	0.0093
4	0.6131	0.4770	0.6181	0.3534	0.3371	0.9497	0.0927	0.0684	0.0789	0.0086
5	0.6783	0.5150	0.6615	0.2834	0.6578	1.1307	0.0709	0.0908	0.0771	0.0114
**average**	0.6469	0.4754	0.6813	0.3902	0.5104	1.0815	0.0802	0.0796	0.0833	0.0084

**Table 7:** MAE obtained with 20% missing data using MICE, AAA and the newly proposed algorithm HAAA.

	**MAE MICE 20% Missing Data**									
**ID#**	**Van**	**Vbn**	**Vcn**	**Vab**	**Vbc**	**Vca**	**Ia**	**Ib**	**Ic**	**PF**
1	19.8495	29.2709	19.7782	33.5372	32.2953	41.2306	0.5143	0.2057	1.2582	0.0032
2	16.1709	26.7847	22.7646	26.1709	36.1606	43.6232	0.6040	0.2416	1.1681	0.0026
3	17.6532	26.4314	21.7054	33.1059	28.5129	42.8703	0.2775	0.1110	0.8885	0.0027
4	15.8018	28.1184	20.4781	25.7729	36.6865	38.5835	0.3310	0.1324	0.7742	0.0023
5	18.9570	29.9625	20.0141	35.7336	28.8170	36.8561	0.2886	0.1155	1.4832	0.0034
**average**	17.6865	28.1136	20.9481	30.8641	32.4944	40.6328	0.4031	0.1612	1.1144	0.0028
	**MAE AAA Algorithm 20% Missing Data**									
**ID#**	**Van**	**Vbn**	**Vcn**	**Vab**	**Vbc**	**Vca**	**Ia**	**Ib**	**Ic**	**PF**
1	0.8996	0.9180	0.8043	1.0513	1.7261	1.4762	0.6694	0.8079	0.6984	0.0014
2	0.8944	0.8669	0.7321	1.4339	1.4942	1.5055	0.8636	0.8248	0.8678	0.0015
3	0.8441	0.8711	0.6416	1.5295	1.6559	1.6439	0.8366	0.8794	0.7254	0.0016
4	0.7184	0.8919	0.8057	1.4782	1.8988	1.4632	0.8848	0.8029	0.9249	0.0018
5	0.7525	0.8642	0.7273	0.8251	0.9897	1.4285	0.7865	0.8989	0.8785	0.0019
**average**	0.8218	0.8824	0.7422	1.2636	1.5529	1.5035	0.8082	0.8428	0.8190	0.0016
	**MAE NEW Algorithm 20% Missing Data**									
**ID#**	**Van**	**Vbn**	**Vcn**	**Vab**	**Vbc**	**Vca**	**Ia**	**Ib**	**Ic**	**PF**
1	0.1137	0.1810	0.1239	0.6595	0.7907	1.2953	0.7436	0.7280	0.7254	0.0011
2	0.1130	0.1702	0.1327	0.7933	0.6688	1.3942	0.7912	0.6542	0.7816	0.0012
3	0.1132	0.1547	0.1472	0.4827	0.5122	1.2889	0.7063	0.7437	0.8055	0.0012
4	0.0984	0.1658	0.1326	0.4718	0.4555	1.1273	0.8911	0.6175	0.7862	0.0014
5	0.1177	0.1846	0.1300	0.5498	0.9242	1.3971	0.6435	0.8116	0.7326	0.0015
**average**	0.1112	0.1713	0.1333	0.5914	0.6703	1.3006	0.7551	0.7110	0.7663	0.0013

**Table 8:** RMSE obtained with 20% missing data using MICE, AAA and the newly proposed algorithm, HAAA for the case in which there is correlation in the missingness of data.

	**RMSE MICE 20% Missing Data**									
**ID#**	**Van**	**Vbn**	**Vcn**	**Vab**	**Vbc**	**Vca**	**Ia**	**Ib**	**Ic**	**PF**
1	24.4908	43.2588	47.5089	65.2910	52.2601	68.8207	0.7967	1.1136	1.8646	0.1084
2	26.4326	61.1208	42.9646	57.2527	61.4969	71.0171	1.2437	0.9490	1.2511	0.1627
3	33.4717	61.9103	48.7703	55.0477	52.4416	106.0520	1.0616	0.8542	1.6592	0.0826
4	35.8859	50.2750	30.4530	54.3050	38.1316	68.8768	0.6942	0.6224	2.6681	0.1056
5	44.4516	52.6348	40.4844	90.2603	29.1994	58.1034	1.2995	0.7163	1.6545	0.1789
**average**	32.9465	53.8399	42.0362	64.4314	46.7059	74.5740	1.0191	0.8511	1.8195	0.1276
	**RMSE AAA Algorithm 20% Missing Data**									
**ID#**	**Van**	**Vbn**	**Vcn**	**Vab**	**Vbc**	**Vca**	**Ia**	**Ib**	**Ic**	**PF**
1	1.9878	1.7252	1.8032	2.8163	4.1109	5.3343	0.1437	0.1631	0.2222	0.0103
2	2.4359	1.6420	2.4058	3.0857	3.1677	4.3184	0.2665	0.2600	0.2786	0.0056
3	1.3291	1.4607	2.4013	2.3697	2.7676	5.6870	0.1173	0.1481	0.2999	0.0073
4	1.8910	1.3804	1.1758	3.7502	2.4218	3.9166	0.3068	0.2228	0.1346	0.0070
5	1.3798	1.8577	1.5385	2.1441	2.9301	3.6357	0.1499	0.2220	0.1604	0.0108
**average**	1.8047	1.6132	1.8649	2.8332	3.0796	4.5784	0.1968	0.2032	0.2191	0.0082
	**RMSE New Algorithm 20% Missing Data**									
**ID#**	**Van**	**Vbn**	**Vcn**	**Vab**	**Vbc**	**Vca**	**Ia**	**Ib**	**Ic**	**PF**
1	1.0966	1.6848	1.3832	0.5570	0.5171	0.5755	0.1876	0.1183	0.1214	0.0024
2	1.2825	1.1124	1.2688	0.4565	0.6296	1.1775	0.1510	0.1534	0.1903	0.0028
3	0.9783	1.2418	1.1965	0.7558	0.8273	0.8882	0.1609	0.1871	0.1724	0.0034
4	1.2532	1.1211	1.4008	0.6415	0.7131	0.5738	0.1775	0.1299	0.1208	0.0017
5	1.1888	1.4617	1.3264	1.2255	0.7568	0.4553	0.1965	0.2133	0.1078	0.0028
**average**	1.1599	1.3243	1.3151	0.7273	0.6888	0.7341	0.1747	0.1604	0.1425	0.0026

**Table 9:** MAE obtained with 20% missing data using MICE, AAA and the newly proposed algorithm HAAA for the case in which there is correlation in the missingness of data.

	**MAE MICE 20% Missing Data**									
**ID#**	**Van**	**Vbn**	**Vcn**	**Vab**	**Vbc**	**Vca**	**Ia**	**Ib**	**Ic**	**PF**
1	38.1829	54.5223	23.8789	58.9845	38.1634	52.8209	0.8517	0.4084	2.2732	0.0062
2	24.1992	33.7322	43.3651	45.5847	65.1002	63.0445	0.8267	0.4078	2.1729	0.0051
3	27.6520	33.3112	26.5114	46.9331	43.6067	56.4079	0.3682	0.1129	1.0877	0.0053
4	22.4674	51.2556	26.8316	37.0569	45.6670	54.6107	0.4494	0.2114	1.2456	0.0029
5	23.7421	49.7850	32.9160	38.4813	55.2293	65.3112	0.3032	0.1294	2.1787	0.0066
**average**	27.2487	44.5212	30.7006	45.4081	49.5533	58.4390	0.5599	0.2540	1.7916	0.0052
	**MAE AAA Algorithm 20% Missing Data**									
**ID#**	**Van**	**Vbn**	**Vcn**	**Vab**	**Vbc**	**Vca**	**Ia**	**Ib**	**Ic**	**PF**
1	1.3325	1.3119	0.9742	1.8596	2.9608	2.8876	0.9854	1.3046	0.7014	0.0022
2	1.4758	1.2017	1.4369	1.7713	2.1919	1.8424	1.4863	0.8386	1.3763	0.0023
3	0.8996	0.9740	0.7864	2.7649	3.0907	2.8334	1.4497	1.2269	1.3796	0.0029
4	0.7598	1.1519	0.8644	1.7245	3.1458	2.4398	1.1288	1.0362	1.2571	0.0028
5	1.4621	1.3658	0.7819	1.5175	1.3176	1.4345	0.8660	1.6700	1.0700	0.0023
**average**	1.1860	1.2011	0.9687	1.9276	2.5414	2.2876	1.1832	1.2152	1.1569	0.0025
	**MAE New Algorithm 20% Missing Data**									
**ID#**	**Van**	**Vbn**	**Vcn**	**Vab**	**Vbc**	**Vca**	**Ia**	**Ib**	**Ic**	**PF**
1	0.1442	0.2838	0.1714	1.0236	1.5602	2.5453	0.9079	1.1350	1.0716	0.0015
2	0.1183	0.2924	0.1868	0.8085	0.9362	2.0889	1.5490	0.8778	1.0643	0.0021
3	0.1604	0.2698	0.2391	0.5750	0.9770	2.4446	0.9220	1.4670	0.9484	0.0023
4	0.1155	0.1715	0.2262	0.5195	0.7244	2.1240	1.2272	1.2134	1.2432	0.0022
5	0.1373	0.3592	0.1652	0.8841	1.3553	2.4234	0.9504	1.4772	0.7877	0.0023
**average**	0.1351	0.2754	0.1977	0.7622	1.1106	2.3252	1.1113	1.2341	1.0230	0.0021

**Table 10:** MAE and RMSE obtained with 90% missing data in a single column (case of missing information in Van) using MICE, AAA and the newly proposed algorithm HAAA.

ID#	90% Missing Data in One Single Columm					
	MAE			RMSE		
	MICE	AAA	HAAA	MICE	AAA	HAAA
1	87.1375	10.2289	8.9960	103.8879	12.3165	8.7614
2	68.0226	10.1661	8.9438	99.2328	12.8503	8.6589
3	76.3340	10.1900	8.4414	86.4040	11.8568	9.1133
4	67.0027	8.8589	7.1839	90.5413	10.5458	7.9936
5	82.5461	10.5940	7.5248	102.5351	13.6865	9.5870
**average**	76.2086	10.0076	8.2180	96.5202	12.2512	8.8228
